# Respiratory syncytial virus pneumonia in an adult cord blood transplant recipient during the SARS‐CoV‐2 outbreak

**DOI:** 10.1002/jha2.351

**Published:** 2021-12-04

**Authors:** Takaaki Konuma, Masamichi Isobe, Seiko Kato, Satoshi Takahashi, Yasuhito Nannya

**Affiliations:** ^1^ Department of Hematology/Oncology The Institute of Medical Science The University of Tokyo Tokyo Japan; ^2^ Division of Clinical Precision Research Platform The Institute of Medical Science The University of Tokyo Tokyo Japan

A 52‐year‐old man, who 8 months previously received single‐unit cord blood transplantation for acute myeloid leukemia, presented with a 4‐day history of nonproductive cough and sore throat. Chest radiography and computed tomography revealed the presence of bilateral ground glass opacities affecting mainly the upper lobes (Figure 1). Testing for polymerase chain reaction (PCR) of severe acute respiratory syndrome coronavirus 2 (SARS‐CoV‐2) in nasopharyngeal swab was negative despite its pandemic in Japan. An extensive search for other pathogens identified respiratory syncytial virus (RSV) by PCR of a nasopharyngeal swab, confirming the diagnosis of RSV pneumonia, which rarely affects late phase stem cell transplant recipients [1]. Epidemic patterns of infectious viruses other than SARS‐CoV‐2 altered substantially in these 2 years, presumably due to the changes in social activities associated with SARS‐CoV‐2 pandemics [2, 3]. For instance, RSV infection was highly suppressed in 2020 but had a resurgent outbreak in early summer 2021 [4, 5]. Therefore, RSV pneumonia should be considered in immunocompromised adults.

**FIGURE 1:**
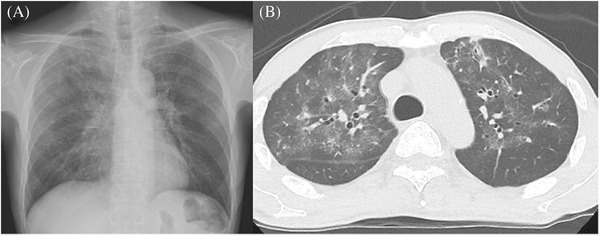
(A) Chest radiography and (B) computed tomography revealed the presence of bilateral ground glass opacities affecting mainly the upper lobes

## CONFLICT OF INTEREST

The authors declare no conflict of interest.

## AUTHOR CONTRIBUTIONS

All authors participated in the care of the patient. Takaaki Konuma wrote the first manuscript draft.
